# The association between blood glucose and oxidized lipoprotein(a) in healthy young women

**DOI:** 10.1186/1476-511X-9-103

**Published:** 2010-09-21

**Authors:** Kazuhiko Kotani, Shingo Yamada, Shuumarjav Uurtuya, Toshiyuki Yamada, Nobuyuki Taniguchi, Ikunosuke Sakurabayashi

**Affiliations:** 1 Department of Clinical Laboratory Medicine, Jichi Medical University, Tochigi, Japan; 2 Central Research Institute, Shino-Test Corporation, Kanagawa, Japan; 3 Saitama Memorial Hospital, Saitama, Japan

## Abstract

**Background:**

Oxidized lipoproteins play important roles in the atherosclerotic processes. Oxidized lipoprotein(a) (oxLp(a)) may be more potent in atherosclerotic pathophysiology than native Lp(a), a cardiovascular disease-relevant lipoprotein. Increased blood glucose concentrations can induce oxidative modification of lipoproteins. The aim of this study was to investigate the association between circulating oxLp(a) and cardiometabolic variables including blood glucose in healthy volunteers within the normal range of blood glucose.

**Methods:**

Several cardiometabolic variables and serum oxLp(a) (using an ELISA system) were measured among 70 healthy females (mean age, 22 years).

**Results:**

Lp(a) and glucose were significantly and positively correlated with oxLp(a) in simple correlation test. Furthermore, a multiple linear regression analysis showed oxLp(a) to have a weakly, but significantly positive and independent correlation with only blood glucose (*β *= 0.269, *P *< 0.05).

**Conclusions:**

These results suggest that increased glucose may enhance the oxidization of Lp(a) even at normal glucose levels.

## Background

Oxidized lipoproteins, such as oxidized low-density lipoprotein (oxLDL), are involved in the atherosclerotic processes [1]. Lipoprotein(a) (Lp(a)) contains low-density lipoprotein (LDL)-like moieties, in which the apoB-100 component is covalently linked to the unique glycoprotein apolipoprotein(a) (apo(a)), and it is also a risk factor for cardiovascular disease (CVD) [2,3]. Oxidized Lp(a) (oxLp(a)) has been observed in human atherosclerotic sites in arteries and blood [4]. Earlier experimental studies have shown that oxLp(a) can add specific atherogenic properties to native Lp(a) [5-10]. On the other hand, there are few clinical studies on oxLp(a) [4,11,12].

This is, in part, due to the lack of specific assays that can be easily used in clinical settings. A few different ELISA systems to detect circulating oxLp(a) have been reported with limited clinical findings [4,11,12]. In one assay system developed by us, complicated hypertensives showed higher serum oxLp(a) levels than normotensives, despite the fact that there was no difference in native Lp(a) levels between hypertensives and normotensives [4]. In another assay system, the patients with end-stage renal disease treated with continuous ambulatory peritoneal dialysis demonstrated higher plasma oxLp(a) levels than those without renal disease [11]. In an additional assay system, the CVD patients exhibited higher plasma levels than the healthy volunteers [12]. Taken together experimental findings, oxLp(a) may be more potent than native Lp(a) in atherosclerotic pathophysiology. Accumulating *in vivo *evidence to elucidate the significance of oxidization in Lp(a) is therefore crucial in better understanding the mechanistic roles of Lp(a) and oxLp(a) on atherogenesis.

Reactive oxygen species have been shown to occur in relatively high blood glucose concentrations, and the oxidative stress promotes glycation-linked oxidative modification of lipoproteins [13,14]. This can result in the vascular damage; thus, the relationship between glucose and oxidized lipoproteins is of interest. This study examined the association between blood concentrations of oxLp(a) and cardiometabolic variables including glucose among healthy subjects.

## Methods

A total of 70 Japanese young women (mean age; 22.2 years, range; 18-26 years) were enrolled in this study. The eligibility criteria were: 1) within normal fasting plasma glucose levels (< 5.56 mmol/L) and without a history of diabetes mellitus, 2) without obesity (body mass index [BMI] < 25 kg/m^2^), hypertension (systolic/diastolic blood pressure [SBP/DBP] < 140/90 mmHg) and lipid abnormality (LDL-cholesterol [LDL-C] < 3.64 mmol/L, triglyceride [TG] < 1.7 mmol/L or high-density lipoprotein-cholesterol [HDL-C] > 1.04 mmol/L), 3) without pregnancy, 4) neither regularly drank alcohol nor smoked, 5) drug-free (including oral contraceptives and over-the-counter drugs such as antioxidant agents), 6) without any history of cardio/cerebrovascular, thyroid, kidney or liver diseases. The Jichi Medical University ethics committee approved the study and each subject gave informed consent.

The BMI, seated SBP/DBP in the upper-arm, fasting levels of blood glucose and lipids/lipoproteins, including Lp(a) and oxLp(a), were measured. Glucose and lipids were measured enzymatically, and serum C-reactive protein (CRP) was measured by a latex agglutination immunoassay. Plasma insulin was measured by an enzyme immunoassay. Serum Lp(a) was measured by an ELISA system (Shino-test Co. Ltd., Japan) [4]. Serum oxLp(a) level was also quantified by a sandwich ELISA using the oxLp(a)-specific monoclonal antibody (161E2) as both the solid-phase antibody and the detecting capture antibody, as previously described [4]. This monoclonal antibody has been proven to react with only oxLp(a), not native Lp(a) and LDL [4]. Especially, the antibody detects a specific epitope peptide of 9 residues (Arg-Asn-Pro-Asp-Ala-Val-Ala-Ala-Pro) from kringle-IV type-2 of apo(a), which appears on the Lp(a) particle with oxidative modification [4]. In the measurements, serum samples were placed in each well of the Nunc-polystyrene microplates coated with anti-oxLp(a) monoclonal antibody. The plates were incubated for 1-hour at room temperature, and after washing, were incubated for 1-hour at room temperature with anti-oxLp(a) monoclonal antibody labeled with peroxidase conjugate. After washing, 3,3',5,5'-tetra-methylbenzidine was added to each well, and the enzymatic reaction was thereafter carried out for 30-minutes at room temperature. After stopping the reaction, the absorbance was read at 450 nm. The concentration of oxLp(a) was calculated based on the concentration of bovine serum albumin (BSA)-peptide that contributed to 16 peptides per 1 molecule of BSA at a standard [4]. The intraassay and interassay coefficients of variation were 1.2% and 5.0%, respectively.

In this study, the cardio-ankle vascular index (CAVI) [15] and carotid arterial intima-media thickness (IMT) [16] were added as measures considered to reflect the presence of atherosclerotic manifestations. The CAVI was measured using oscillometric technology in the supine position (Fukuda Denshi Co. Ltd., Japan) [15]. The IMT of the common carotid arteries was measured ultrasonographically by a 10-MHz linear type B-mode probe (Aloka Co. Ltd., Japan). The IMT, bilaterally measured in segments free of plaque (one at the thickest site and another at two other points [1-cm upstream and 1-cm downstream from the thickest site]), was averaged in the 3-measurements.

The data are expressed as the mean ± standard deviation or median plus interquartile range. The simple correlations between oxLp(a) and the other variables were examined by Pearson's correlation test as well as a multiple linear regression analysis controlled for the variables. The values of TG, insulin, Lp(a) and oxLp(a) were calculated after a log-transformation because of the skewed distribution. A *P *< 0.05 was considered significant.

## Results

**Figure 1:**
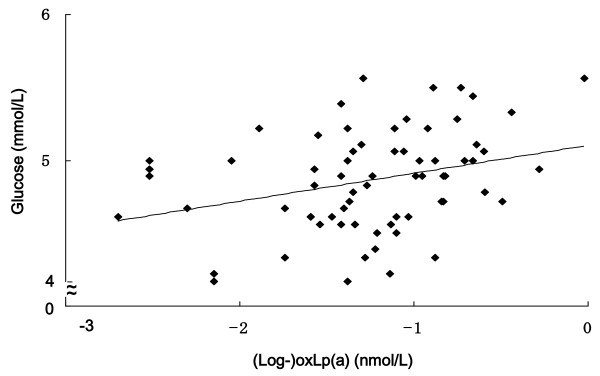
**Correlation between glucose and oxidized lipoprotein(a) (oxLp(a))**.

The subjects' characteristics are presented in Table 1 and the correlations between oxLp(a) and the other variables are listed in Table 2. A simple correlation test showed in addition to a significantly positive correlation between Lp(a) and oxLp(a), that glucose was significantly and positively correlated with oxLp(a) (Figure 1). On the other hand, glucose was non-significantly correlated to Lp(a) (*r *= 0.190, *P *= 0.115). OxLp(a) was non-significantly correlated to CAVI (*r *= 0.196, *P *= 0.103) and IMT (*r *= - 0.041, *P *= 0.734). Lp(a) was non-significantly correlated with either CAVI (*r *= - 0.014, *P *= 0.911) or IMT (*r *= - 0.074, *P *= 0.643). A multiple linear regression analysis adjusted for age and the other cardiometabolic variables (BMI, SBP, DBP, LDL-C, HDL-C, TG, glucose, insulin and CRP) revealed only glucose levels to be independently significantly and positively correlated with those of oxLp(a).

**Table 1:** Clinical characteristics of the study subjects

Variable	mean/median
Age (years)	22.2 ± 1.9
Body mass index (kg/m2)	20.1 ± 2.2
Systolic blood pressure (mmHg)	108.3 ± 8.2
Diastolic blood pressure (mmHg)	65.5 ± 6.0
LDL-cholesterol (mmol/L)	2.54 ± 0.59
HDL-cholesterol (mmol/L)	1.69 ± 0.24
Triglyceride (mmol/L)	0.63 (0.48-0.89)
Glucose (mmol/L)	4.85 ± 0.34
Insulin (μU/mL)	0.77 (0.58-0.90)
C-reactive protein (mg/dL)	0.06 ± 0.12
Lipoprotein(a) (mmol/L)	0.10 (0.06-0.27)
Oxidized lipoprotein(a) (nmol/L)	0.06 (0.03-0.13)
Cardio-ankle vascular index	5.4 ± 0.5
Carotid intima media thickness (mm)	0.40 ± 0.07

LDL, low-density lipoprotein; HDL, high-density lipoprotein. Data are shown as the mean ± standard deviation or the median plus interquartile range.

**Table 2:** Correlations of oxidized lipoprotein(a) with the other variables

Variable	r (P value)	β (P value)
Age (years)	0.044 (0.719)	- 0.012 (0.931)
Body mass index (kg/m2)	- 0.012 (0.919)	0.026 (0.858)
Systolic blood pressure (mmHg)	0.035 (0.774)	- 0.082 (0.647)
Diastolic blood pressure (mmHg)	0.139 (0.250)	0.175 (0.333)
LDL-cholesterol (mmol/L)	- 0.060 (0.623)	- 0.124 (0.381)
HDL-cholesterol (mmol/L)	0.173 (0.153)	0.174 (0.245)
Triglyceride (mmol/L)	0.058 (0.635)	0.147 (0.307)
Glucose (mmol/L)	0.313 (0.008)**	0.269 (0.048)*
Insulin (μU/mL)	0.138 (0.255)	0.049 (0.736)
C-reactive protein (mg/dL)	0.029 (0.810)	0.025 (0.847)
Lipoprotein(a) (mmol/L)	0.565 (< 0.0001)**	-

LDL, low-density lipoprotein; HDL, high-density lipoprotein. *r*: Pearson's correlation coefficient between oxidized Lipoprotein(a) and another variable, *β*: multiple linear regression coefficient in a multivariate analysis adjusted for age and the following variables: body mass index, systolic blood pressure, diastolic blood pressure, LDL-cholesterol, HDL-cholesterol, triglyceride, glucose, insulin, C-reactive protein. Triglyceride, insulin, lipoprotein(a) and oxidized lipoprotein(a) were calculated after a log-transformation, because of their skewed distributions. Significance level: * *P *< 0.05, ** *P *< 0.01.

## Discussion

In this study, neither Lp(a) nor oxLp(a) were associated with the measures of atherosclerotic manifestations such as CAVI and IMT (these measures showed normal levels). This result was expected, because our study population was prepared to demonstrate a good state of vascular health, in order to observe the relationship of the blood cardiometabolic variables with oxLp(a) as simply as possible. Furthermore, we found a relatively good correlation between Lp(a) and oxLp(a). This seems to be plausible because native Lp(a) is a source of oxLp(a), while an earlier study reported that in normotensives, the correlation between Lp(a) and oxLp(a) might not always be significant [4]. This may partly stem from the differences in studied populations.

A more important finding is the significant positive and independent correlation between glucose and oxLp(a), not Lp(a), in the absence of defined hyperglycemia. Although the correlation level was not necessarily strong, this finding is nevertheless noteworthy due to the fact that it was obtained from a strict study population of the abovementioned otherwise healthy subjects. Therefore, the present finding suggests that Lp(a) may be oxidized with an increase in blood glucose concentration even within the normal range of glucose. Increased blood glucose can promote the oxidative capacity of modification of proteins and lipids [13,14]. This may explain the previous observation that oxLDL exists with high glucose concentrations [13,14,17]. Further studies are required to determine whether lower blood glucose concentrations are preferable even at normal glucose levels in order to avoid the oxidization of Lp(a).

The detection methodology of oxLp(a) remains a debatable point. The respective ELISA systems, developed so far, have different features [4,11,12]. One assay system employs an antibody to LGE_2 _([R]-acetyl-9[R]-formyl-12[S]-hydroxy-5[Z],10[E]-heptadeca-dienoic acid)-protein pyrroles, and the system may only examine an early stage of oxidization of lipoproteins [11]. Another assay system uses autoantibodies against oxLp(a), and these antibodies possibly cover various epitopes [12]. One merit of our system is that a peptide epitope, which appears in the oxidative modification of Lp(a), is clearly defined to detect oxLp(a) [4]. However, in addition to the fact that the measureable amount of oxLp(a) is not very much in this method, there is an opinion that the present system may not distinguish the number of Lp(a) particles from the number of modified kringle-IV repeats, because the antibody used in this system reacts with the epitope on kringle-IV type-2. Although earlier studies have shown that there is less or, if possible, not so large influence of the kringle-IV repeat numbers on the measured oxLp(a) levels [4], we acknowledge the need for continuous studies on the association between apo(a) size isoform heterogeneity and oxLp(a) assay systems.

There are several limitations associated with this study. The study population was relatively small. Moreover, it will be important to investigate various populations besides healthy populations as ours. The cross-sectional design did not clearly elucidate the cause-and-effect on the results. For instance, the positive correlation between glucose and oxLp(a) may not only be the result of oxidation of lipoproteins by glucose but also a reflection of functional aspects of Lp(a) (i.e., Lp(a) can reportedly act as a scavenger (transporter) of oxidized lipids derived from oxLDL [18]). In addition, the residual confounders that may affect the oxidization of lipoproteins, but were not included in our present study (i.e., dietary factors), should also be considered. Therefore, more studies with larger and various samples, prospective designs and the inclusion of many confounders are called for when conducting future studies.

## Conclusions

In short, oxLp(a) showed a significantly positive correlation with blood glucose among healthy young women. Lipoprotein(a) may be oxidized with increased glucose even within the normal glucose levels. Further research is warranted to confirm this theory.

## Competing interests

The authors declare that they have no competing interests.

## Authors' contributions

All authors contributed the intellectual development of this work, and approved the final manuscript. KK and SU collected the samples. KK, SY, TY and TN analyzed the samples and data. KK, SY and SU wrote the draft paper. KK, SY and TY searched the literature, and TN provided critical corrections to the manuscript.
